# Bloodstream infections at a tertiary hospital in the Gambia - a one-year retrospective study

**DOI:** 10.1186/s12879-025-10533-1

**Published:** 2025-02-05

**Authors:** Paul Rahden, Ebrima Barrow, Haddy Bah, Sheikh Omar Bittaye, David Nygren, Abdoulie Badjan

**Affiliations:** 1 Department of Internal Medicine, Edward Francis Small Teaching Hospital, Banjul, FC4C+WW4 The Gambia; 2 Department of Implementation Research, Bernhard-Nocht-Institute for Tropical Medicine, Bernhard-Nocht-Str. 74, 20359 Hamburg, Germany; 3 Microbiology Unit, Department of Laboratory Medicine, Edward Francis Small Teaching Hospital, Banjul, FC4C+WW4 The Gambia; 4 School of Medicine and Allied Health Sciences, University of The Gambia, FC4F+J3C, Independence Drive, Banjul, The Gambia; 5 Division of Infection Medicine, Lund University, Lund University, BMC, B14, 221 84 Lund, Sweden; 6 Department of Infectious Diseases, Skåne University Hospital, Ruth Lundskogs gata 3, Malmö, 20502 Sweden

**Keywords:** Bloodstream infections, Bacteraemia, Antimicrobial Resistance, Sub-saharan Africa, The Gambia, Low resource setting

## Abstract

**Introduction:**

Antimicrobial resistance is a significant global health concern, particularly in western Sub-Saharan Africa. This study describes causes of bloodstream infections and antimicrobial resistance at a tertiary hospital in The Gambia.

**Methods:**

This retrospective analysis included all blood cultures performed at the Edward Francis Small Teaching Hospital, Banjul, The Gambia, from September 2022 to August 2023. Blood culture positivity-rates and pathogens were described. Antimicrobial susceptibility testing was performed using disk diffusion following the Clinical & Laboratory Standards Institute guidelines.

**Results:**

A total of 645 patients had blood cultures drawn during the study period with 260 (40%) positive results. Contaminants were identified in 28 cases (4%). The majority were drawn from neonatal or paediatric patients (360/645, 56%) and overall primarily in intensive care units (406/645, 63%). The median age was 3 years (interquartile range 0–31 years) and 46% were female. The most common pathogens were *Staphylococcus aureus* 106/260 (41%), *Klebsiella spp.* 41/260 (16%), other bacteria within the *Enterobacterales* order 33/260 (13%), *Pseudomonas spp.* 22/260 (8%) and *Acinetobacter spp.* 19/260 (7%). Methicillin-resistant *S. aureus* (MRSA) was seen in 34/58 (59%) tested. Extended-spectrum beta-lactamases (ESBL) were seen in 36/40 (90%) of *Klebsiella spp*. and in 16/28 (57%) of other bacteria within the *Enterobacterales* order. Acquired antibiotic resistance, beyond wild-type, was reported in 17/20 (85%) of *Pseudomonas spp*. and 16/19 (84%) of *Acinetobacter spp*.

**Conclusion:**

Overall, blood culture positivity rates were high, indicating restrictive testing suggesting that sample collection were restricted to mainly critically ill, neonatal or paediatric patients. Nonetheless, our data suggests a high proportion of bloodstream infections due to multi-drug resistant pathogens, including MRSA and ESBL-*Enterobacterales*. Importantly, generalisability of findings beyond this tertiary hospital setting remains restricted. However, our findings demonstrate a need for improved diagnostic stewardship and ongoing surveillance to provide robust evidence-based data to inform antimicrobial resistance interventions.

**Supplementary Information:**

The online version contains supplementary material available at 10.1186/s12879-025-10533-1.

## Introduction

### Background

Antimicrobial resistance (AMR) is a growing public health concern and the Western Sub-Saharan Region has been estimated to suffer from the highest burden worldwide [1, 2]. Scarcity of diagnostics, effective drugs, and reliable surveillance systems increase the impact of AMR in Sub-Saharan Africa [3, 4].

In The Gambia, most microbiological surveillance data has been described by the Medical Research Council Gambia (MRCG), a research institute and secondary health care facility in Fajara, outside of the capital Banjul. Previously, they have described *Streptococcus pneumoniae*, *Staphylococcus aureus (S. aureus)*, *Escherichia coli (E.coli)* and *Non-typhoidal Salmonella* (*NTS*) as the most prevalent causes of bacteraemia [5]. Resistance rates to first and second-line antibiotics recommended in national and African CDC treatment guidelines, such as penicillin, trimethoprim-sulphonamide or third generation cephalosporins in lower respiratory tract infections, have been low in studies from MRCG [6, 7]. Methicillin resistant *S. aureus* (MRSA) has been described in 1–13% of infections caused by *S. aureus* while presence of extended spectrum beta-lactamases (ESBL) in *Klebsiella spp.* and *E. coli* has been described in up to 35% and 23% based on cultures on varying patient samples (e.g. urine, blood, faeces) [5, 8, 9]. However, regional data from selected patient groups, especially paediatric and neonatal patients, have shown much higher resistance rates, with ESBL rates in *E. coli* and *Klebsiella spp.* infections ranging up to 50 and 100%, respectively [10–12]. Conversely, in one Gambian study investigating ESBL-carriage in faeces, a relatively low rate of 5% was described among food handlers [13]. As there is no continuous national surveillance and reporting system in The Gambia, the burden of antimicrobial resistance among hospitalised patients and in the community largely remains unknown with varying rates previously reported.

Additionally, the healthcare system in The Gambia faces several challenges related to potential drivers of AMR. These challenges include limited availability of microbiological testing, stock-outs of basic consumables, mis- and overuse of antibiotics, lack of trained staff and laboratory facilities [3, 4, 14, 15]. Furthermore, while paediatric care for children < 5 years of age is free and subsidised for children from 5 to 14 years of age, most health care interventions in The Gambia require out-of-pocket payments for diagnostics and therapeutics for adults [16].

In this study, we aimed to identify the most common causes of bloodstream infections in children and adults at the only tertiary hospital in The Gambia over a 12-months period. Additionally we sought to evaluate antimicrobial resistance patterns among the most commonly identified pathogens to assess the burden of AMR at the country’s main teaching and referral hospital.

## Methods

### Study design and setting

This retrospective study was conducted at the Edward Francis Small Teaching hospital (EFSTH) in Banjul, The Gambia. EFSTH was the only tertiary hospital in the country offering specialist care, dialysis and intensive care, in addition to around the clock services for laboratory requests. It had a capacity of 550 beds and served as the only referral centre for the 2.7 million inhabitants of the country with approximately 16,000 hospitalised patients yearly. We extracted baseline characteristics (age, gender, and ward), bacterial findings and antimicrobial susceptibility testing (AST) in patients who had blood cultures drawn over a 12-month period from September 2022 to August 2023. Data was retrieved from paper-based records from the Department of Microbiology at EFSTH. No clinical data was available in these records, including no information on previous or current treatment. Data was pseudonymized and then analysed using Microsoft Excel (Version 16.83) and Stata Statistical Software (Release 17). Graphs were designed using datawrapper software (https://www.datawrapper.de/).

### Study participants

Case eligibility was any patient with a blood culture drawn from September 2022 to August 2023 at the EFSTH. No patients were excluded. Samples were drawn in all departments of emergency and acute medicine, surgery, internal medicine, paediatrics, paediatric surgery, obstetrics and gynaecology. In general, patients 14 years or younger were defined and treated as children (≤ 28 days as neonates, > 28 days to 14 years as paediatric patients) while patients aged 15 years or older were treated in adult departments at EFSTH. In the case of missing data on age, patients were categorised as neonatal, paediatric or adult based on which ward they were admitted to. Neonates were generally admitted to the NICU and not to regular paediatric wards if hospital admission was necessary as no other neonatal wards were available.

### Microbiological methods

Specimen collection procedures followed established protocols for the collection of blood cultures. Samples were drawn via venepunctures from new sites and preceded by disinfection of the access sites. Ideally, eight to ten millilitres of blood were collected and inoculated into a set of BD BACTEC™ PLUS culture bottles each (one aerobic and one anaerobic) for adults and for children a minimum of 0.5 ml blood was drawn to a maximum of 5 ml in one BD BACTEC™ PLUS paediatric culture bottle. Due to financial constraints and limited availability of blood culture resources, only one pair of blood cultures was drawn. Repeated blood cultures, e.g. in *S. aureus* bacteraemia, were not common practice at the hospital.

Blood culture samples were transported to the microbiology unit by staff members after collection. Samples received between 8:00 AM and 8:00 PM were immediately placed in the BD BACTEC™ FX40 incubator upon arrival. Samples received during nights were handled by the on-call lab staff and, if immediate processing was not possible, were stored at 35–36℃ until 8:00 AM. Blood culture bottles were incubated for five days. A gram stain was performed from positive bottles for preliminary identification followed by sub-culturing onto blood, Chocolate and MacConkey agar. All agars were produced in-house following standardised recommendations of the manufacturer. Blood agar was prepared using 5% human blood obtained from expired blood donations from the hospital blood bank. Sheep blood was not routinely available.

Blood and MacConkey plates were incubated aerobically whereas Chocolate plates were incubated in 5% CO_2_. All were kept at 35–36℃ and reviewed after 24 h when growth was compared across the plates.

#### Species identification

Species identification was based on the following procedures. Most gram-positive organisms grew on blood and chocolate media, while gram-negative cocci may grow on either or both of blood and chocolate agar. Gram-negative bacilli would grow on all three plates [17]. In case of polymicrobial growth across the various plates or growth patterns that did not conform to the above, contamination was suspected and the plates were discarded if confirmed. Phenotypic species identification was performed using manual biochemical methods. In gram-positive cocci, identified through gram stain and morphology appearance, a catalase test was performed using 1% hydrogen peroxide. If positive, the isolate was further speciated using Staphaurex™ Plus Latex Agglutination Test. This classified isolates as either *S. aureus* or coagulase negative *Staphylococcus*. Did clinical information suggest further need, such as a concomitant finding of *S. saprophyticus* in urine culture and hence a suspected urinary tract infection as cause of bacteraemia, a novobiocin disc test was performed to differentiate *S. epidermidis* from *S. saprophyticus*. For catalase negative gram-positive cocci, the haemolytic pattern was considered. β-haemolytic colonies on blood agar were subjected to a bacitracin disk susceptibility test with Group A streptococci, specifically *S. pyogenes*, exhibiting sensitivity while Group B (*S. agalactiae*), Group D (including Enterococci *spp*.) were expected to demonstrate resistance. For resistant β-haemolytic cocci a CAMP test was done to confirm Group B streptococci. For α-haemolytic organisms an optochin disc test was performed to differentiate *Streptococcus pneumoniae* from other α-haemolytic Streptococci. Catalase negative gram-positive cocci that were neither identified as Group A or B Streptococci were tested for growth in NaCl 6.5% media to differentiate Enterococcus species, which grew in this medium, from non-enterococcal Group D Streptococci.

Gram-negative bacilli identified as fermenters from MacConkey agar were phenotypically identified using triple sugar iron (TSI), 2% urea and citrate media. In these instances identification was generally done to genera level. If the identification process gave ambiguous results or subsequent antimicrobial testing showed a multidrug resistant organism BIOMÉRIEUX API^®^ ID 20E was used for further species identification if available. Non-fermenting negative bacilli were screened using the oxidase test. Oxidase-negative isolates were then tested using TSI, 2% urea, and citrate media, following the same protocol as above.

*Acinetobacter spp.* were identified by the presence of oxidase-negative, gram-negative coccobacilli, which exhibited an alkaline/alkaline reaction on TSI agar, were citrate positive, and urea and indole negative. *Salmonella spp.* were identified through biochemical analysis, such as the BIOMÉRIEUX API^®^ ID 20E system. Serotyping was performed using polyvalent and monovalent antisera to detect bacterial O and H antigens.

*Pseudomonas spp.* isolates (non-fermenters) were identified through colonial morphology, gram stain and oxidase reaction (oxidase positive). Finally gram-negative coccobacilli, if isolates grew only on chocolate agar, were screened using factor X and V on nutrient agar to speciate *Haemophilus spp*. Suspected *Neisseria spp.* on the other hand were identified based on gram stain, colonial morphology, and a positive oxidase test.

#### Antimicrobial susceptibility testing

AST was done per Clinical and Laboratory Standards Institute (CLSI) guidelines with Kirby-Bauer disk diffusion [18]. Inoculum for plating were prepared using 0.5 MacFarland saline suspension of the target organism. For the identification of MRSA, a cefoxitin test was performed. ESBL-production was defined as resistance to third generation cephalosporins in pathogens of the *Enterobacterales* group using double disc synergy testing. Acquired antimicrobial resistance in *Acinetobacter spp*. or *Pseudomonas spp.* was defined as any additional acquired resistance beyond wild-type susceptibility [18]. In accordance with breakpoints from the CLSI guidelines, this was defined as any resistance to ciprofloxacin, aminoglycoside (gentamicin), piperacillin-tazobactam, imipenem-cilastatin, meropenem, or ceftazidime for *Pseudomonas spp*. and any resistance to imipenem-cilastatin, meropenem, aminoglycoside (gentamicin), ciprofloxacin, or trimethoprim-sulphonamide for *Acinetobacter spp*.

Isolates identified as coagulase negative* Staphylococci*, *Bacillus spp.*, *Micrococcus spp.* and coryneform bacteria were regarded as contaminants unless they were isolated in two or more independent blood culture specimens from the same patient and clinical data was suggestive of pathological growth. In those cases they were included as a pathogen.

In the case of polymicrobial bacteraemia, each distinct bacterial species identified was recorded in terms of species distribution of positive cultures. However, if the polymicrobial growth was attributed to contaminants, the entire sample was classified as a single contaminant.

In general, diagnostic testing was impacted by stock-outs and supply shortages at the hospital resulting in intermittently limited availability of kits for species identification and antibiotic discs for AST.

### Outcomes and variables

Blood culture results were presented per pair for adults and per single bottle for paediatric patients. Cultures were considered positive if at least one pathogen was identified that was not classified as contaminant. The primary analysis focused on blood culture positivity rates and distribution of pathogens identified grouped in neonatal, paediatric or adult patients, highlighting the five most common pathogens identified. The secondary analyses highlighted resistance patterns in the five most commonly identified pathogens and investigated differences in distribution of isolates cultured from patients in ICU vs. non-ICU.

### Data analysis

Descriptive statistics were mainly reported. For categorical variables, counts and percentages were presented with Fischer’s exact test used for statistical comparisons. For continuous variables, median and interquartile range (IQR) were presented. The study sample size was determined by the total amount of blood cultures performed during the study period. No formal power analysis was performed a priori as the primary aim of the study was to provide descriptive statistics and not statistical comparison.

## Results

### Participants baseline characteristics

In total, 645 patients had blood cultures performed. Their baseline characteristics are summarised by age group in Table 1. 268 (42%) of the patients were neonatal, 92 (14%) paediatric and 277 (43%) were adults. 406 (63%) of the overall samples were drawn in an ICU, with an overwhelming majority of samples in neonates, 265/268 (99%), drawn in the NICU. The remaining three neonatal samples were drawn in the paediatric emergency unit.

**Table 1:** Baseline characteristics and blood culture positivity rates of patients across age groups

Characteristics	Neonatal ≤ 28 d *n* = 268	Paediatrics > 28 d-15 y *n* = 92	Adults ≥ 15 y *n* = 277	Total *n* = 645
Age, median (IQR)	5 (3–10) d	4.0 (0.7-7) y	35 (27–53) y	3 (0–31) y
Female, %^*^	119/262 (45%)	32/92 (35%)	142/277 (51%)	297/639 (46%)
Blood culture drawn in ICU, %^*^	265/268 (99%)	24/92 (26%)	117/277 (42%)	406/645 (63%)
Blood culture positivity rate, %^*, **^	140/268 (58%)	33/92 (36%)	84/277 (30%)	260/645 (40%)

^*^Some patients had missing data on gender (*n* = 6) or age group (*n* = 8). If a definite allocation was not possible samples were included in the total column but not in the age specific columns

^**^Excluding contaminants

## Main results

### Most common pathogens identified in blood cultures

Of the 645 patients, 260 (40%) had bloodstream infections, excluding 28 (4%) patients with positive cultures classified as contaminants. Blood culture positivity rate was higher among neonatal patients (140/268, 58%), compared to paediatric (33/92, 36%, *p* = 0.008) or adult patients (84/277, 30%, *p* < 0.001). No patient was identified as being positive twice during the same hospitalisation, equating all episodes of bacteraemia as unique.

**Fig. 1:**
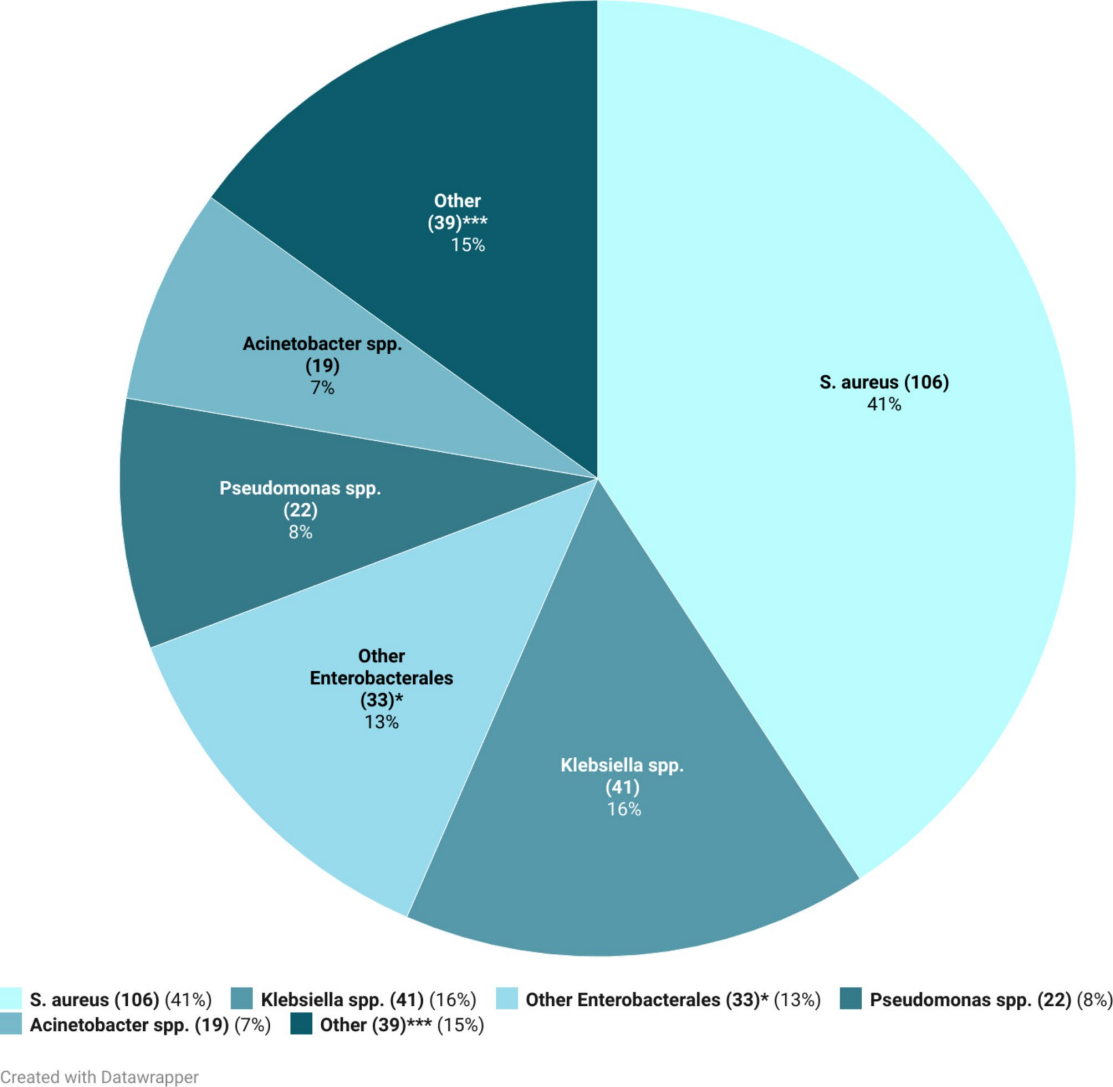
Distribution of pathogens isolated from positive blood cultures (defined as identification of at least one pathogen in a pair of cultures for adults or a single bottle for paediatrics). * Other Enterobacterales include all identified pathogens in the Enterobacterales group, except Klebsiella spp., which is reported separately since it was the most common finding of this group. ** Contaminants include results labelled as contaminants, typically coagulase-negative Staphylococci. *** e.g. other include Streptococcus spp., Enterococci spp., Candida spp

The most common pathogens identified were *S. aureus* with 106/260 cases (41%), and *Klebsiella spp*. with 41/260 cases (16%). Other pathogens of the *Enterobacterales* group identified were seen in 33/260 cases (13%), *Pseudomonas spp*. in 22/260 cases (8%) and *Acinetobacter spp*. in 19/260 cases (7%). *Streptococci spp*. or *Salmonella spp.* were only identified in eight and zero cases respectively. One fungal bloodstream infection was identified (*Candida spp.)*. Findings are displayed in Fig. 1.

When bloodstream infections were grouped as neonatal, paediatric or adult, *S. aureus* bacteraemia was more commonly seen among paediatric and adult cases, while neonatal *S. aureus* bacteraemia was slightly less common (Table 2). Differences were smaller for gram-negative pathogens. Contamination was not seen among paediatric patients, whereas it was relatively common in both neonates (16/268, 6%) and adults (12/277, 4%). Given the lack of clinical data, it was not possible to ascertain whether these represented catheter-associated infections or truly represented skin contaminants.

**Table 2:** Blood culture findings of patients with blood stream infection summarised by age groups

Characteristics	Neonatal ≤ 28 d*	Paediatrics > 28 d-15 y*	Adults ≥ 15 y*
*Staphylococcus aureus* (%)	45/140 (32%)	16/33 (48%)	43/84 (51%)
*Klebsiella spp*. (%)	24/140 (17%)	3/33 (9%)	14/84 (17%)
*Other Enterobacterales* (%)	22/140 (16%)	4/33 (12%)	7/84 (8%)
*Pseudomonas spp.* (%)	13/140 (9%)	5/33 (15%)	4/84 (5%)
*Acinetobacter spp. (%*)	16/140 (11%)	0/33 (0%)	3/84 (4%)
Other	20/140 (14%)	5/33 (15%)	10/84 (12%)

* *n* = 3 patients had missing data on age group. If a definite allocation was not possible samples were included in the total count but not in the age specific columns. One blood culture had a polymicrobial infection

When cases with bloodstream infections were grouped as ICU or non-ICU regardless of age group (Table 3:1–2), blood culture positivity rates were higher in the ICU (183/406 (45%) vs. non-ICU 77/239 (32%) respectively, Fischer’s exact test, *p* = 0.002). *S. aureus* represented the most common finding in both groups, yet was more prevalent in non-ICU patients (41/77, 53%) compared to ICU-patients (65/183, 36%, *p* = 0.009). Rates of MRSA were higher in ICU patients, yet not statistically significant (*p* = 0.16). The distribution and resistance rates of Gram-negative pathogens were similar overall, except for *Acinetobacter spp*. which was solely identified in ICU patients (19/183, 10%).

**Table 3:** 1–2: Blood culture findings and resistance rates of patients with bloodstream infections from ICU and Non-ICU wards

**Table 3:1** **Species**	***Staphylococcus aureus***	***Klebsiella spp.***	**Other** *** Enterobacterales***	***Pseudomonas*** ***spp.***	***Acinetobacter*** ***spp.***	**Other**
ICU, n/total (%)	65/183 (36%)	31/183 (17%)	26/183 (14%)	16/183 (9%)	19/183 (10%)	26/183 (14%)
Non-ICU, *n*/total (%)	41/77 (53%)	10/77 (13%)	7/77 (9%)	6/77 (8%)	0/77 (0%)	15/77 (19%)
*P*-value**	**0.009**	0.46	0.31	1	**0.0013**	
**Table 3:2** **Resistance rates**	***Staphylococcus aureus***, **MRSA**	***Klebsiella spp.***, **ESBL**	**Other*Enterobacterales***,*** ESBL***	***Pseudomonas spp.***, **acquired resistance beyond wild-type**	***Acinetobacter spp.***, **acquired resistance beyond wild-type**	
ICU, *n*/total (%)	25/38 (66%)	27/30 (90%)	13/23 (57%)	12/14 (86%)	16/19 (84%)	
Non-ICU, *n*/total (%)	9/20 (45%)	9/10 (90%)	3/5 (60%)	5/6 (83%)	0/0 (0%)	
*P*-value**	0.16	1	1	1	1	

*No resistance rates are reported for other pathogens

** Fischer’s exact test was used for statistical comparisons of ICU vs. non-ICU rates

#### Results in resistance patterns

Among the five most common pathogens identified, AST was evaluated if performed. Of a total of 106 cases of *S. aureus*-bacteraemia, 58/106 (55%) samples had available data on AST. Among these isolates, 34/58 (59%) were defined as MRSA. In the isolates defined as MRSA (*n* = 34), resistance to other non-beta lactam antibiotics were described. Resistance was seen in 21/32 (66%) to macrolides, in 16/17 (94%) to fluroquinolones, in 11/14 (79%) to aminoglycosides, in 16/26 (62%) to tetracyclines and in 5/8 (63%) to trimethoprim-sulphonamide. Resistance to these antibiotics in methicillin-sensitive *S. aureus* was less commonly seen when compared to MRSA (data not shown).

For pathogens of the *Enterobacterales* group, presence of ESBL was investigated. First *Klebsiella spp*. were specifically investigated, since it was the most common gram-negative finding. In 40/41 (98%) isolates, AST were performed. Among these isolates, 36/40 (90%) were defined as ESBL. For *Klebsiella spp*. isolates defined as ESBL, the resistance rates for other antibiotics were investigated and reported if tested. Resistance was seen in 16/28 (55%) for fluoroquinolones, in 20/20 (100%) for trimethoprim-sulphonamide and in 8/11 (73%) for gentamicin. Carbapenems or amikacin were not tested for in any of the *Klebsiella spp*. isolates.

Among other pathogens of the *Enterobacterales* group, 28/33 (85%) were investigated for ESBL. Among these isolates, 16/28 (57%) were defined as ESBL. The resistance rates for other antibiotics were investigated and reported if tested. Resistance was seen in 5/12 (42%) for fluoroquinolones, 10/12 (83%) for trimethoprim-sulphonamide and 7/8 (88%) for gentamicin. Carbapenems were investigated in three isolates, all were susceptible. Amikacin resistance was not tested for.

For *Pseudomonas spp*. 20/22 (91%) were investigated for any acquired resistance beyond wild-type. Acquired resistance were seen in 7/14 (50%) to fluoroquinolones, in 7/11 (64%) to beta-lactam antibiotics and in 9/10 (90%) to gentamicin. Beta-lactams investigated were ceftazidime, for which 7/10 (70%) were resistant, and imipenem-cilastatin for which 0/1 (0%) was resistant. Amikacin resistance was not tested for.

**Fig. 2:**
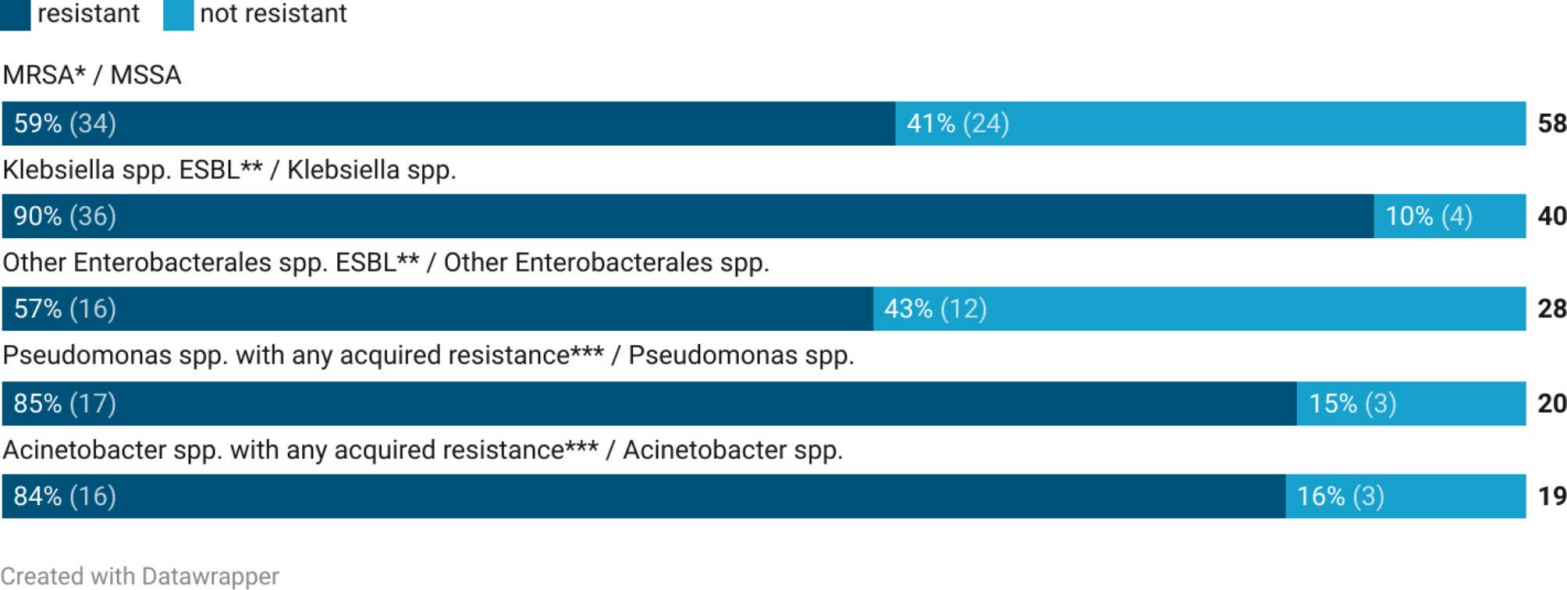
Resistance patterns of the five most common pathogens in patients with positive blood culture results. Total numbers of positive blood cultures results where antimicrobial susceptibility testing was performed are presented at the right of the graph (n) with resistance rates presented for each. Totals of Other Enterobacterales include all Enterobacterales except Klebsiella spp. * MRSA was defined as any resistance to methicillin or similar (cefoxitin test). ** ESBL was defined as any resistance to third generation cephalosporins. ***Defined as any acquired resistance beyond wild-type resistance in Acinetobacter spp. or Pseudomonas spp.

For *Acinetobacter spp*. 19/19 (100%) were evaluated for any acquired resistance beyond wild-type. Acquired resistance were seen in 5/17 (29%) for fluoroquinolones, in 5/14 (36%) for beta-lactam antibiotics, in 13/14 (93%) for trimethoprim-sulphonamide and in 3/3 (100%) for gentamicin. Beta-lactams investigated were ceftazidime, for which 5/14 (36%) were resistant, and imipenem-cilastatin, for which 0/1 (0%) was resistant. Amikacin resistance was not tested for. Resistance patterns for the most common pathogens are summarised in Fig. 2.

## Discussion

Our study describes the blood culture positivity rates, distribution of pathogens and their antimicrobial resistance rates in bloodstream infections at a tertiary hospital in The Gambia over a 12-month period. The study revealed an overall blood culture positivity rate of 40% (260/645). The highest positivity rate was found in neonates and among ICU-patients. The most frequently identified pathogens in descending order were *S. aureus*, *Klebsiella spp*., other *Enterobacterales*, *Pseudomonas spp*. and *Acinetobacter spp*. Among *S. aureus*, MRSA-rates were surprisingly high, as were ESBL-rates in *Klebsiella spp*. and other *Enterobacterales spp*. Similarly, most strains of *Pseudomonas spp*. or *Acinetobacter spp*. had acquired resistance beyond their wild-type susceptibilities.

Previous studies from The Gambia have identified *S. aureus*, *S. pneumoniae*, *E. coli* and non-typhoidal *Salmonella* (*NTS)* as the most prevalent pathogens in bacteraemia [5, 8, 19, 20]. The largest analysis by Darboe et al. highlighted a prevalence of *S. pneumoniae* (25%), *S. aureus* (22%), *E. coli* (11%) and *NTS* (10%) as the most common pathogens found among patients with community-acquired bloodstream infections [5]. In comparison, our analysis revealed a higher rate of *S. aureus* (41%). Conversely, a low rate of *Streptococcus spp.* (8/260, 3%) and no cases of *Salmonella spp.* were identified in our study, whereas *Klebsiella spp.* and *Enterobacterales* except *Klebsiella spp.* were seen in 41/260 (16%) and 33/260 (13%) respectively. In addition, pathogens more associated with nosocomial infections (*Pseudomonas spp.* (22/260, 8%) and *Acinetobacter spp.* (19/260, 7%)) were relatively common in our study.

High blood culture positivity rates were seen in all age groups indicating restrictive testing and antimicrobial resistance rates identified were emphasising the threat of bloodstream infections in our setting [2, 21, 22]. Efficacious outpatient antibiotic treatment, no or late microbiological assessments as well as lack of appropriate testing options might be causes of these findings [11, 14]. In our study, the high positivity rate observed in neonates are particularly concerning as they are at risk for poor outcome in infections due to poor immune response. The high resistance rates found possibly contribute to the high infant mortality rate (34/1,000 live births) and children under five mortality rate (48/1,000 live births) in The Gambia [23]. Additionally, our findings are in line with previous data that have suggested a higher proportion of gram-negative bacteraemia in low-income settings where particularly infants are up to five times more likely to suffer from gram-negative bloodstream infections [24].

In comparison to previous regional data, resistance rates presented in this study are higher [5, 8, 9]. Darboe et al. reported MRSA in less than 1% of bloodstream infections with *S. aureus* and ESBL in 35% of *Klebsiella spp.* and 23% of *E.coli* cases [8]. On the contrary, in our study 59% of cases with *S. aureus* bloodstream infections were MRSA, whereas ESBL was identified in 90% of cases with *Klebsiella spp.* and 57% of cases with other *Enterobacterales.* It is likely that patients presenting to the tertiary hospital (EFSTH) and in whom blood cultures were performed were selected, possibly with prior antibiotic exposure or referral due to complex or severe conditions. The fact that 64% of overall samples were sent from ICU or NICU settings is indicative of the selection of critically ill patients. To assess for potential selection bias we compared the distribution of pathogens and AMR between ICU and non-ICU patients. However, the differences in resistance rates did not reach statistical significance, possibly due to insufficient power.

### Limitations

The study limitations are mainly due to the retrospective single centre design. Selection bias of severe cases and restrictive testing at this tertiary hospital have been highlighted. Likely, this impacts both species distribution and resistance rates. As mentioned, a vast majority of patients who had blood cultures drawn were admitted to an ICU and the majority of patients were neonatal or paediatric which impacts interpretation. Thus, the generalisability of our findings beyond the tertiary hospital setting in this Western African country is limited. Furthermore, clinical information on onset and type of symptoms, previous or current treatment or outcomes were not available. Since data were extracted from microbiological records and no electronic medical record system is available at EFSTH that links results to individual patients, findings could not be linked to case files retrospectively. Additionally, diagnostic precision was impacted by stock-outs due to constraints in funding and supply shortages at the hospital. This results in limited availability of kits for species identification as well as antibiotic discs for AST. Consequently, our analysis focus on the most commonly encountered pathogens and antibiotics. In this regard, we reported other *Enterobacterales* as a group as stock-outs made definite species identification uncertain in some cases. Also, anaerobic incubation conditions were imperfect due to the lack of an anaerobic chamber as well as gas packs to create an anaerobic atmosphere in anaerobic jars. To our knowledge, there is no published data on anaerobic bloodstream infections in The Gambia. Therefore, further evaluation is necessary to understand their clinical impact. Additionally, it is possible that insufficient availability of antibiotic discs could lead to selective usage, i.e. prioritising patients who are most critically ill, which might overestimate resistance rates. This further limit generalisability of our findings. Despite these limitations, the study findings outline high antibiotic resistance rates in this setting providing valuable insights into the challenge of AMR at EFSTH and represents the first study examining neonates and critically ill to this extent in The Gambia.

### Future prospects

Given the findings of our analysis, future studies are required to better understand the distribution and resistance patterns of both community-acquired and nosocomial bloodstream infections and to describe the clinical impact of the high antimicrobial resistance rates here reported. Therefore, a prospective study is underway to assess microbiological data combined with clinical information and outcomes. In addition, antimicrobial stewardship programs are currently being implemented. These efforts could help continuously inform tailored guidelines for the local context to improve rational use of antimicrobials and improve management of patients with bloodstream infections.

## Conclusion

This study revealed high proportions of culture-confirmed bloodstream infections due to multi-drug resistant pathogens, including MRSA and ESBL-*Enterobacterales*, based on data from primarily critically ill, neonatal and paediatric patients at the only tertiary hospital in The Gambia. These findings highlight the need for a regional surveillance system of AMR along with increased availability of microbiological diagnostics and the rational use of antimicrobials to mitigate the impact of bloodstream infections due to drug resistant pathogens.

## Electronic supplementary material

Below is the link to the electronic supplementary material.

Supplementary Material 1

## Data Availability

The datasets generated and analyzed during the current study are available from the corresponding author on request. This data availability is consistent with ethical guidelines and institutional policies concerning privacy and confidentiality.

## Abbreviations

AMR: Antimicrobial Resistance
AST: Antimicrobial Susceptibility Testing
CLSI: Clinical & Laboratory Standards Institute
E. coli: Escherichia coli
EFSTH: Edward Francis Small Teaching Hospital
ESBL: Extended–Spectrum Beta Lactamase
ICU: Intensive Care Unit
ICQ: Interquartile Range
MRCG: Medical Research Council Gambia
MRSA: Methicillin Resistant Staphylococcus aureus
NTS: Non–typhoidal Salmonella
NICU: Neonatal Intensive Care Unit
S. aureus: Staphylococcus aureus
TSI: Triple Sugar Iron
